# Sex Differences in Step Count-Blood Pressure Association: A Preliminary Study in Type 2 Diabetes

**DOI:** 10.1371/journal.pone.0014086

**Published:** 2010-11-22

**Authors:** Priya Manjoo, Lawrence Joseph, Louise Pilote, Kaberi Dasgupta

**Affiliations:** 1 Department of Medicine, Division of Clinical Epidemiology, McGill University Health Centre, Montreal, Canada; 2 Department of Medicine, Vancouver Island Health Authority, Victoria, Canada; Pennington Biomedical Research Center, United States of America

## Abstract

**Background:**

Walking and cardiovascular mortality are inversely associated in type 2 diabetes, but few studies have objectively measured associations of walking with individual cardiovascular risk factors. Such information would be useful for “dosing” daily steps in clinical practice. This study aimed to quantify decrements in blood pressure and glycated hemoglobin (A1C) per 1,000 daily step increments.

**Methodology/Principal Findings:**

Two hundred and one subjects with type 2 diabetes underwent assessments of step counts (pedometer-measured), blood pressure, A1C and anthropometric parameters. Due to missing data, the final analysis was conducted on 83 women and 102 men, with a mean age of 60 years. Associations of daily steps with blood pressure and A1C were evaluated using sex-specific multivariate linear regression models (adjusted for age, ethnicity, and BMI). Potential sex differences were confirmed in a combined model (women and men) with interaction terms. Mean values for daily steps, blood pressure, A1C and BMI were 5,357 steps/day; 137/80 mm Hg; 7.7% and 30.4 kg/m^2^ respectively. A 1,000 daily step increment among women was associated with a −2.6 (95% CI: −4.1 to −1.1) mm Hg change in systolic and a −1.4 (95% CI: −2.2 to −0.6) mm Hg change in diastolic blood pressure. Among men, corresponding changes were −0.7 (95% CI: −2.1 to 0.7) and −0.6 (95% CI: −1.4 to 0.3) mm Hg, respectively. Sex differences were confirmed in combined models. Step counts and A1C did not demonstrate clinically important associations.

**Conclusions/Significance:**

A 1,000 steps/day increment is associated with important blood pressure decrements among women with type 2 diabetes but the data were inconclusive among men. Targeted “dose increments” of 1,000 steps/day in women may lead to measurable blood pressure reductions. This information may be of potential use in the titration or “dosing” of daily steps. No associations were found between step count increments and A1C.

## Introduction

Physical activity is associated with lower rates of all-cause mortality in the general population [1]. However, physical activity promotion is an underutilized therapeutic strategy in patients with cardio-metabolic disease. Walking is a cheap, easily accessible means of increasing physical activity, and studies indicate that it is the preferred form of activity among overweight adults with type 2 diabetes [2]. In the Nurses' Health Study, among over 5,000 women with diabetes, those in the highest quartile for self-reported walking were 34% less likely to have died up to 8 years later [3]. In the all-male Health Professionals' Follow-up Study, among over 3,000 men with diabetes diagnosed after 30 years of age, men in the highest walking quintile were 43% less likely to have died up to 14 years later [4]. Similarly, the National Health Interview Survey (n = 2,896) demonstrated that walking more than two hours per week was associated with a more than 34% reduction in both all-cause and vascular disease mortality up to 9 years later among men and women with diabetes [5].

In healthy adults aged 26–80 years, lower levels of pedometer or accelerometer-based categories of physical activity have been shown to be associated with a higher odds or prevalence of adverse cardio-metabolic risk factors [6], [7]. In addition, a previous systematic review and meta-analysis determined that pedometer-based programs may lead to higher daily step counts and lower blood pressure levels in several clinical populations, particularly when a specific target is provided (e.g., 10,000 steps/day) [8]. Although this supports the potential utility of “walking prescriptions”, “dose-response curves” have not been defined in the literature. Moreover, there is a lack of studies assessing the association of daily step counts with individual cardiovascular risk factors, particularly in a treated clinical cohort: the existing studies are small, with inconsistent results and different measures of walking [9]–[12]. Therefore, the present study aims to contribute to the evidence base for what constitutes an important “daily step increment”.

Well-designed observational studies evaluating the associations of objectively measured walking with individual cardiovascular risk factors are an important preliminary stage in determining whether walking is of measurable benefit in an already-treated population with type 2 diabetes. By providing estimates of step-related improvements in individual cardiovascular risk factors, this information can be used to facilitate the development of “walking prescriptions”. The availability of step counters or pedometers for real-time measurement of walking makes this strategy both practical and economically feasible. A widely used classification scheme for daily step counts initially proposed by Tudor-Locke and Bassett [13] and subsequently revised [14] categorizes individuals achieving <5,000 steps/day as sedentary; 5,000–7,499 as low active; 7,500–9,999 as somewhat active; 10,000–12,499 as active; and ≥12,500 as highly active [13]–[15]. This study proposes to build on the existing body of evidence related to pedometer-measured physical activity through measurement of the association of daily steps with specific vascular risk factors. Blood pressure and glycated hemoglobin (abbreviated A1C, a marker the degree of glycemic control over the preceding 3 months) are established vascular risk factors and important therapeutic targets for reducing cardiovascular mortality, stroke and micro-vascular complications in patients with type 2 diabetes [16].

Specifically, the present study aims to quantify the changes in systolic blood pressure, diastolic blood pressure, and A1C associated with each 1,000 daily step increment among women and men with type 2 diabetes. At a moderate walking pace, 1,000 steps per day may be achieved in approximately ten minutes; thus a 1,000 steps/day increment arguably represents a feasible “dose increase in daily steps,” keeping an eye to eventual clinical application [17]. We performed sex-specific analyses given that previous studies have suggested that women reduce their blood pressure somewhat more consistently with exercise training than men [18]–[20].

## Methods

Data for the present study were derived from a cohort study designed to assess for seasonal differences in step counts and A1C. Data collection procedures for our cohort have been detailed previously but are summarized here [21]. Participants presented for quarterly assessments over one year (once per season) performed by research personnel at the Division of Internal Medicine, Montreal General Hospital site of the McGill University Health Centre. The main analyses reported here are based on data collected during and immediately after the first study centre visit. These are cross-sectional data as the goal was to capture associations across a wide range of daily step counts and cardiovascular risk factor levels occurring across individuals rather than within individuals. In a secondary analysis, we also examined within-individual associations using the available longitudinal data.

### Participants

Two hundred and one adults followed for type 2 diabetes were recruited through McGill University affiliated outpatient clinics between June 2006 and June 2008 (Montreal, Canada). To ensure the accuracy of the pedometer measurements, participants were required to have a normal gait and a BMI ≤40 kg/m^2^ [22], [23]. Patients with stable chronic conditions were permitted to enroll. Women who were pregnant or planning pregnancy were excluded.

### Procedures

Demographic information (sex, date of birth, ethnicity and level of education) and a detailed medical history (including duration of diabetes and current medication use) were obtained via questionnaire and interview at each study centre visit. Data were collected on cardiovascular risk factors, physical activity including daily steps, anthropometric parameters and dietary information, as outlined below. All assessments were performed at our study centre at the McGill University Health Centre (Division of Internal Medicine). For the purpose of our analyses, ‘smokers’ included persons who quit smoking less than a year prior to entry into the study.

### Cardiovascular Risk Factors

Blood pressure measurements were taken in the left arm using an appropriate-sized cuff (Omron HEM 747 IC blood pressure monitor) after a 15 minute rest period. A1C was measured from blood samples collected at each visit using high-pressure liquid chromatography.

### Daily Step and Physical Activity Measurement

Daily step counts were measured using Yamax SW-200 pedometers. The accuracy and reliability of this device has been previously demonstrated [24]. At each of the four visits, participants were provided with three pedometers, labeled A, B and C. All pedometers were fitted with a snap-on plastic cover that concealed the viewing window which was sealed using an acetate security seal (Novovision). This seal was placed along the margin of the pedometer and the cover so that any attempt to tamper with the seal was therefore evident. Participants were instructed to wear pedometer A during waking hours for 7 consecutive days, after which it was removed and replaced by pedometer B for another 7 consecutive days. Pedometers A, B and C were then mailed to our study centre in a pre-paid, pre-addressed, padded courier envelope. Pedometer C served to measure (false) steps registered during the mailing process (“postman steps”). These postman steps were subtracted from the step count of each of Pedometers A and B and the sum of the remaining steps were averaged over the total time period worn (i.e., 14 days). The Short Last 7 Days Self-Administered format of the International Physical Activity Questionnaire (IPAQ) was used to assess self-reported levels of overall physical activity and to calculate metabolic equivalents per week [25].

### Anthropometric Measurements and Dietary Intake

Weight and height were assessed to the nearest tenth of a kilogram (SECA 882 electronic scale) and tenth of a centimeter (SECA 214 stadiometer) respectively, with the subject wearing light clothing and with shoes removed. Waist circumference was measured midway between the iliac crest and the lower rib margin. Hip circumference was measured at the point of greatest posterior extension of the buttocks. The waist-to-hip ratio was calculated by dividing the waist circumference by the hip circumference. BMI was calculated by dividing the weight in kilograms by the square of the height in meters. The Quebec Food Frequency Questionnaire (FFQ), previously validated in a sample of adults in the Montreal region, was used to gather dietary information [26].

#### Ethics

Procedures were approved by the Institutional Review Boards (IRB) of McGill University and participating institutions (McGill University Health Centre, Sir Mortimer Davis Jewish General Hospital, and Centre de Santé et de Services Sociaux de la Montagne). All study participants provided written informed consent prior to the clinical assessments.

#### Statistical methods

Participants were classified by daily step quartiles based on their first visit data (Table 1). Characteristics were computed by daily step quartile as appropriate (mean and standard deviation for continuous variables; proportions for categorical variables).

**Table 1:** Participant Characteristics (n = 188) By Daily Step Quartiles.

	Step-Count Quartiles			
Characteristic	≤3,512	3,513–5,357	5,358–7,399	≥7,400
Women, No. (%)	22 (46)	25 (53)	21 (45)	18 (38)
Age, mean (SD), y	65 (11)	63 (9)	57 (10)	57 (9)
Diabetes duration, mean (SD), y	11 (8)	10 (8)	9 (8)	9 (8)
White, No. (%)	37 (78)	33 (70)	28 (60)	30 (64)
Completed High School, No. (%)	39 (83)	42 (89)	42 (89)	39 (83)
Cardiovascular Disease, No. (%)	12 (26)*	8 (17)*	7 (15)*	7 (15)*
Current tobacco use, No. (%)	3 (6)†	6 (13)	4 (9)†	5 (11)‡
Insulin use, No. (%)	21 (45)	16 (34)	12 (26)	14 (30)
≥2 Anti-hypertensives, No. (%)	35 (74)	27 (57)	25 (53)	9 (19)
No Anti-hypertensives, No. (%)	6 (13)	5 (11)	10 (21)	17 (36)
Anthropometric Parameters				
Waist Circumference, mean (SD), cm	107.2 (12.1)	103.6 (12.1)	100.5 (14.8)	97.1 (12.5)
Hip Circumference, mean (SD), cm	112.8 (10.7)	112.2 (11.8)	110.9 (12.6)	105.0 (10.9)
Waist-to-Hip Ratio, mean (SD)	0.95 (0.07)	0.92 (0.07)	0.90 (0.07)	0.92 (0.09)
Body Mass Index, mean (SD), kg/m^2^	31.3 (5.1)	31.7 (5.0)	30.1 (6.7)	28.6 (5.4)
Cardiovascular Risk Factors				
Glycated Hemoglobin A1C, mean (SD), %	8.0 (1.9)	7.4 (0.8)	7.6 (1.2)	7.4 (1.3)
Systolic Blood Pressure, mean (SD), mmHg	144 (20)	138 (15)	136 (17)	131 (15)
Diastolic Blood Pressure, mean (SD), mmHg	82 (11)	80 (11)	80 (10)	79 (10)

* Data for 2 subjects missing;

† Data for 1 subject missing;

‡ Data for 4 subjects missing.

Multivariate linear regression was applied to these data to evaluate the associations of daily steps with systolic and diastolic blood pressure and A1C in separate models with adjustment for potential confounders. Each model was constructed separately for women and men. Models combining data from women and men were also examined, with an interaction term between sex and daily steps, to verify sex differences suggested by sex-specific models. Residual plots of the most representative models were examined to verify that the assumptions of linear regression were met. We examined potential confounders by comparing the beta-coefficients of the determinant variable of interest across models containing various combinations of potential confounding variables. Covariates having the strongest impact on this beta-coefficient were identified as statistically important confounders and retained in the final model. These analyses were conducted using the R statistical package, version 2.8.0 (R Foundation for Statistical Computing, Vienna, Austria).

Although our primary analysis was based on study participants' first visit data, each participant had up to four data points over the course of the year. We used a two-level hierarchical modeling on these data to examine within-individual associations of blood pressure (systolic and diastolic) and A1C with daily steps. The first level of this model included linear regression models of each outcome within each subject over time. At the second level of these models, the slopes from the first models were regressed against potential predictors. Previous analyses of our data demonstrated that habitual walking patterns differ by season [27]. In keeping with these findings, we tested season (fall-winter versus spring-summer) as a potential confounder in our hierarchical modeling. Hierarchical modeling was performed using winBUGS [28].

#### Sample Size

It was determined that a sample size of 160 would allow us to examine up to 8 variables in a given model with reasonable accuracy [29]. In a previous study, adherence for subjects required to wear a pedometer for 12 consecutive weeks was 72% [30]. Since our protocol required 2 week periods, we estimated that our shorter period of monitoring would lend itself to a higher adherence rate and anticipated an 80% adherence rate. A final sample size was selected to accommodate for up to 20% missing information. It was therefore anticipated that with recruitment of 200 individuals (100 men and 100 women) we would retain at least 160 individuals.

## Results

Two hundred and one individuals (106 men, 95 women) completed a first assessment. Thirteen participants were excluded because of missing A1C, blood pressure readings or pedometer data. The analyses presented were conducted on 188 participants (86 women, 102 men) for whom the data were complete. First visit assessments were conducted throughout the year (30% in fall, 20% in winter, 25% in spring and 25% in summer). Women comprised 46% of the cohort. The mean age of the population was 60.3 years, their average duration of diabetes was 9.6 years, and 68% of the cohort was Caucasian. Approximately 23% had stable cardiovascular disease with a four-fold higher prevalence among the men compared with the women (30% versus 8%). Participants were obese (mean BMI of 30.3 kg/m^2^) and applying the classification scheme developed by Tudor-Locke and Bassett, 44% were ‘sedentary’, 32% were ‘low-active’, 20% were ‘somewhat active’, 4% were ‘active’ and no study participant was ‘highly active’ [13]–[15]. The average A1C and systolic blood pressure were above the 2008 Canadian Diabetes Association (CDA) recommended targets.

**Figure 1:**
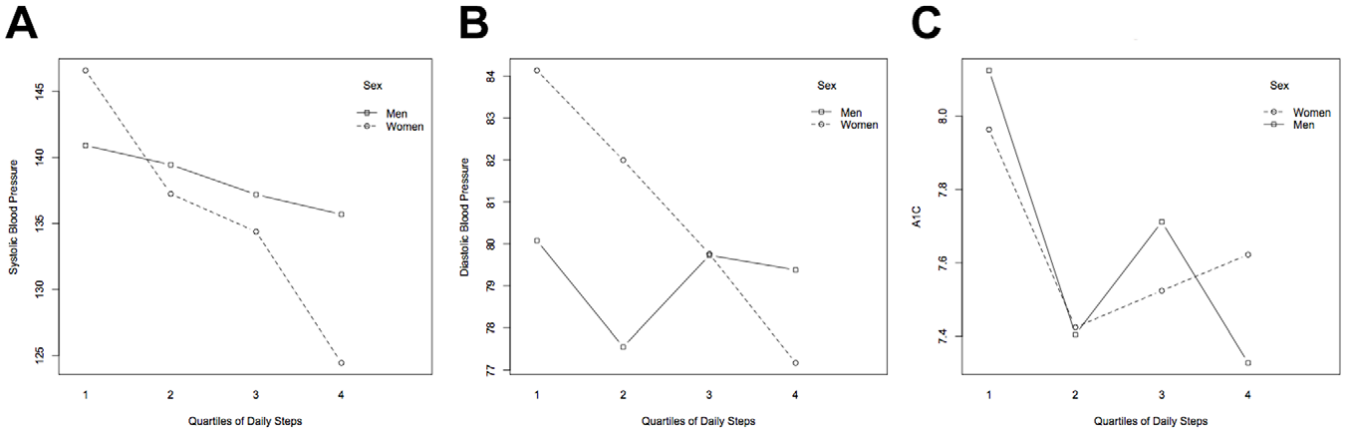
Mean systolic blood pressure (A), diastolic blood pressure (B), and A1C (C) by daily step quartile in women and men treated for type 2 diabetes.

The mean systolic blood pressure, diastolic blood pressure and A1C were highest among participants in the lowest quartile of daily steps, and lowest in the highest quartile of daily steps (Table 1; Figure 1A and 1B). Systolic blood pressure was lowest in the first daily step quartile and highest in the fourth daily step quartile (Table 1). More individuals in the lower daily step quartiles reported use of anti-hypertensive medication use. The largest decrement in A1C was noted between the first and second quartile of daily step counts in both women and men. Diastolic blood pressure and A1C values were more similar from the second through the fourth daily step quartiles.

Model comparisons suggested that age and ethnicity were important confounders across all models tested. Additionally, BMI was a confounder of the association of daily steps with blood pressure and waist-to-hip ratio was a confounder of the association of daily steps with A1C. After adjustment for these confounders, sex-specific analyses (Table 2) indicated that each 1,000 daily step increment was associated with a −2.6 (95% CI: −4.1, −1.1) mm Hg change in systolic and a −1.4 (95% CI: −2.2, −0.6) mm Hg change in diastolic blood pressure among the women. Data regarding these associations among men, though suggestive of an inverse association, were inconclusive. In fully adjusted models, there did not appear to be a clinically important association of walking with A1C in either women or men (Table 2). In multivariate models including data from both women and men, the beta-coefficient for the “sex*daily steps” interaction term suggested an important sex difference (2.3 (95% CI: 0.4 to 4.1)) for systolic but not diastolic blood pressure (0.8 (95% CI: −0.2 to 1.9)).

**Table 2:** Change in Blood Pressure and A1C per 1,000 Daily Step Increment among Women and Men.

	Mean Change (95% CI)	
Characteristic	Women	Men
Systolic blood pressure*, mm Hg	−2.6 (−4.1 to −1.1)	−0.7 (−2.1 to 0.7)
Diastolic blood pressure*, mm Hg	−1.4 (−2.2 to −0.6)	−0.6 (−1.4 to 0.3)
HbA1c†, %	−0.05 (−0.2 to 0.1)	−0.1 (−0.2 to 0.0)

* Models adjusted for age, ethnicity and BMI;

† Models adjusted for age, ethnicity and waist-to-hip ratio.

Hierarchical modeling of the longitudinal data demonstrated a clinically important within-individual inverse association of daily steps with systolic blood pressure among women, specifically during the fall-winter period;: a 1,000 daily step increment was associated with a (−1.4mm Hg (95% CI: −2.3, −0.4)) change in systolic blood pressure (Table 3). The data were inconclusive among the men. There was no significant within-individual association between daily steps and A1C. Although season was an important confounder in these longitudinal analyses, it was not found to be a confounder in our primary cross-sectional analyses.

**Table 3:** Hierarchical Modeling of Longitudinal Data, Demonstrating Seasonal Changes in Blood Pressure per 1,000 Daily Step Increment, among Women and Men.

	Mean (95% CI)	
Characteristic	Winter-Fall	Spring-Summer
Women		
Systolic blood pressure, mm Hg	−1.4 (−2.3 to −0.4)	0.6 (−0.3 to 0.15)
Diastolic blood pressure, mm Hg	0.0 (−0.5 to 0.6)	0.5 (−0.04 to 1.1)
Men		
Systolic blood pressure, mm Hg	−0.3 (−1.1 to 0.4)	0.2 (−0.6 to 1.0)
Diastolic blood pressure, mm Hg	−0.3 (−0.8 to 0.2)	0.2 (−0.3 to 0.7)

All hierarchical models adjusted for age, ethnicity and BMI.

## Discussion

Our analyses suggest that habitual walking decreases blood pressure among persons with type 2 diabetes. A 1,000 daily step increment was associated with as much as a 2.5 mm Hg lower systolic and a 1.4 mm Hg lower diastolic blood pressure among women treated for type 2 diabetes. In a longitudinal “within-individual” analysis, a 1,000 daily step increment was associated with a 1.4 mm Hg lower systolic blood pressure among women (in the fall and winter months). Daily steps were not as strongly associated with blood pressure in the men. Finally, our analyses did not demonstrate a clinically important association between daily steps and A1C in either women or men treated for type 2 diabetes. Clearly, further study is required to confirm these preliminary findings and to verify thresholds of benefit. Nonetheless, our findings indicate that daily step dose increments of 1,000 steps/day could lead to clinically important blood pressure decrements in women with type 2 diabetes.

Pedometer-based intervention trials in type 2 diabetes have achieved increases in daily step counts [31], [32]. The First Step program was the largest and longest such trial in diabetes patients (24 weeks; n = 42) and demonstrated increases of 3,370 steps per day in the intervention arm compared to a 657 daily step decrease in the control arm at 16 weeks. There was an associated −2.5 mm Hg (SD 13.9) change in systolic blood pressure in the intervention arm but a 0.7 mm Hg (SD 13.1) change in the control group. Although the sample size was insufficient to demonstrate a statistically significant difference between the two arms, this study supports the potential effectiveness of pedometer interventions [31]. Our findings suggest that these interventions might be of greatest benefit in controlling cardiac risk factors among women. Across a variety of clinical populations, pedometer-based interventions have been associated with increases of 2,000 to 3,000 steps per day [8]. Applied to our data, these interventions might be associated with reductions in systolic and diastolic blood pressure of 5 to 7.5 mm Hg and 2.8 to 7.2 mm Hg respectively, among women with type 2 diabetes. Blood pressure reductions of this magnitude are clinically important and have been shown to be associated with significant reductions in diabetes-related outcomes such as myocardial infarction and micro-vascular complications [33].

The stronger association of physical activity with blood pressure among the women in this study is consistent with other data showing greater physical activity-associated reductions in blood pressure among women compared with men [18], [19]. These sex differences raise the possibility that walking might be a more effective modifier of cardiovascular risk among the women in our cohort and may have contributed to their lower prevalence of cardiovascular disease despite their higher obesity prevalence (Table 1). Alternatively, since men have a fourfold greater prevalence of heart disease, their individual cardiovascular risk factors might be more established or more aggressively controlled with medications and therefore less modifiable by walking. Further study examining these sex-differences and exploring possible explanations could offer useful insights into the mechanisms responsible for the disparity in cardiovascular risk between women and men.

Our analyses also demonstrated a stronger association between blood pressure and daily steps in the fall and winter compared with the spring and summer in women with type 2 diabetes, in the within-individual analyses. On average, the blood pressure was higher and exercise levels lower in the fall-winter seasons than the spring-summer months. This observed seasonal difference may therefore be reflecting a greater sensitivity of higher blood pressures to daily steps, or a threshold effect, such that an increase in daily step counts may have greater benefits at the lower levels of activity in the fall and winter months.

Our findings support the need for a large pedometer-based intervention trial that examines the impact of 1,000 daily step increments on blood pressure in type 2 diabetes. Furthermore, since our study population is restricted to patients with diabetes being treated at tertiary care facilities, future studies in a less selected population may have greater generalisability. While our findings indicate a stronger association of step counts and blood pressure in women with type 2 diabetes, a larger study may have confirmed an association in men, albeit likely with a “dose” greater than 1,000 steps/day. We acknowledge that step counts may include not only walking but all step-related physical activity such as dancing or stair-climbing, although walking is the most frequent type of physical activity reported by diabetes patients.

Our study is limited by its cross-sectional nature, and as such the associations described cannot be assumed to be causal. Furthermore, we acknowledge the potential for walking pace and the intensity of walking to impact the cardiovascular risk factors evaluated. Randomized control studies employing strategies to account for the pace of walking are needed to confirm these associations and to define dose-response thresholds of benefit in populations for which its use is intended. Community-based studies should also be performed to validate the efficacy of pedometer-based “walking prescriptions”.

Despite these limitations, we believe that our findings are a critical first step in establishing dose-response curves for step counts with blood pressure in patients treated for diabetes and could facilitate the development of individual exercise prescriptions that provide patients with more tangible goals for physical activity. Given the economic feasibility and acceptability of walking in sedentary populations, a prescription of daily steps may prove to be an effective and accepted therapeutic intervention for improving control of cardiovascular risk factors such as blood pressure.
